# Analysis of risk factors for complications after flap reconstruction of head and neck cancer and construction and validation of predictive models

**DOI:** 10.3389/fonc.2025.1580393

**Published:** 2025-12-02

**Authors:** Yakun Fang, Jinlei Fan, Chao Yan

**Affiliations:** 1 Department of Obstetrics, Qingdao Hospital, University of Health and Rehabilitation Sciences (Qingdao Municipal Hospital), Qingdao, China; 2 Department of Radiation Oncology, Qilu Hospital (Qingdao), Cheeloo College of Medicine, Shandong University, Qingdao, China

**Keywords:** head and neck neoplasms, free flap reconstruction, postoperative complications, logistic regression, random forest algorithm, prediction model

## Abstract

**Objective:**

To analyze the importance ranking of influencing factors of postoperative complications of free flap reconstruction in patients with head and neck cancer by using Logistic regression and random forest algorithm, and to construct and verify the prediction model.

**Methods:**

The research subjects were patients with head and neck tumors who underwent free flap reconstruction in our hospital, The clinical-relevant data of all patients were collected. Patients were randomly divided into training set and validation set at the ratio of 7:3. Univariate and multivariate analyses using Logistic regression were performed to identify independent risk factors for postoperative complications. The random forest algorithm was further used to construct the prediction model, and the performance of the model was verified by receiver operating characteristic curve (ROC) analysis, calibration curve evaluation, and decision curve analysis (DCA).

**Results:**

A total of 341 patients were included in the study, and 82 cases (24.05%) had postoperative complications. Multivariate Logistic regression analysis showed that age, hypertension, operation time, bleeding volume, flap type and flap area were the independent risk factors for complications after free flap reconstruction in patients with head and neck cancer (*P* < 0.05). The contribution magnitudes of each variable obtained from the random forest model was flap resection area, intraoperative bleeding volume, age, operation time, flap type and concomitant hypertension. The calibration curve of the constructed Nomogram model showed that the predicted value was in good agreement with the actual value, and the AUC of the ROC curve was 0.793 (95% *CI*: 0.711–0.874) and 0.788 (95% *CI*: 0.665–0.912), respectively, showing good prediction performance. The DCA analysis indicated that the model had good clinical application value.

**Conclusion:**

Logistic regression and random forest algorithm can effectively analyze the influencing factors of complications after free flap reconstruction in patients with head and neck cancer and construct accurate prediction model. This model can provide a scientific theoretical reference for the prevention of postoperative complications and facilitate the precise optimization of individualized prevention plans based on risk prediction.

## 1 Introduction

Head and neck cancer is a serious threat to human health. Free flap reconstruction, as a common repair method after head and neck cancer resection, is of great significance for the recovery of patients’ physiological function and appearance. However, postoperative complications can affect the surgical results, prolong the hospital stay, increase medical costs, and even endanger the life of the patient. Therefore, identifying the influencing factors of postoperative complications and building an accurate prediction model are crucial for clinical decision-making, early intervention and improvement of patient prognosis (1). Previous studies have shown that complications after free flap reconstruction in patients with head and neck cancer are affected by a variety of factors (2). Individual characteristics of patients, such as age, gender, nutritional status, basic diseases (hypertension, diabetes, cardiovascular disease, etc.), will affect the overall state of the body and the tolerance to surgery. Preoperative tumor-related factors, such as tumor location, size, staging, and pathological type, are related to the type and extent of surgery as well as to the choice of the most suitable flap. Surgical-related factors, including operation time, bleeding volume during operation, flap type and harvesting method, and vascular anastomosis technology, directly determine the degree of surgical trauma and blood supply of the flap. Postoperative care and monitoring factors, such as whether the drainage is unobstructed, the anti-infection measures are appropriate, and the monitoring frequency of flap blood supply, also play a key role in the occurrence and development of complications (3). While previous studies have highlighted preoperative radiotherapy and nutritional status as risk factors (2, 3), these variables were not analyzed in our study for three reasons: First, the limited number of preoperatively irradiated cases (n=5) precluded meaningful statistical analysis. Second, standardized nutritional parameters (e.g., serum albumin, BMI) were inconsistently documented in retrospective records, compromising data reliability. Third, our single-center design focused primarily on intraoperative predictors, consistent with the study’s primary aim to establish a surgically actionable model. Future prospective studies should incorporate these factors through standardized protocols. Traditional research methods have some limitations in analyzing the relationship between multi-factors and postoperative complications, and it is difficult to fully and deeply reveal the complex correlation. Logistic regression model can handle the relationship between the two categorical variables, screen out the factors that have a significant impact on the postoperative complications from many clinical indicators, and quantify the effect strength, thus laying the foundation for the construction of prediction model (4). As an advanced machine learning method, random forest algorithm can effectively handle the complex data structure, mine the importance of variables, deal with the nonlinear relationship and variable interaction in the data, and accurately rank the importance of each influencing factor (5). In this study, the advantages of Logistic regression and random forest algorithm were combined to comprehensively collect clinical data of patients, deeply analyze influencing factors, construct and verify prediction model, provide an effective preoperative risk assessment tool for clinical practice, and help improve the treatment effect and safety of free flap reconstruction for head and neck tumors.

## 2 Materials and methods

### 2.1 Subjects

Patients with head and neck cancer who received free flap reconstruction in our hospital from January 2021 to January 2024 were selected. Inclusion criteria: (1) Patients who were diagnosed with head and neck tumor by pathology and underwent free flap reconstruction; (2) Complete clinical data, including preoperative examination, operation records and postoperative follow-up information. Exclusion criteria: (1) Patients who were accompanied with other serious basic diseases such as severe cardiopulmonary failure, severe liver and kidney insufficiency, and could not tolerate the operation; (2) Patients who have received radiotherapy, chemotherapy and other treatment methods before the operation, affecting the surgical evaluation and postoperative recovery; (3) Mental illness history, unable to cooperate with postoperative follow-up and data collection. This study was reviewed and approved by the Hospital Ethics Committee, and all patients signed informed consent forms.

### 2.2 Data collection

Collect general data of the patient, including age, gender, body mass index (BMI), smoking history, alcohol consumption history, and concomitant underlying diseases (hypertension, diabetes, cardiovascular disease, and pulmonary disease); Preoperative tumor-related information, such as tumor location (mouth, throat, neck, etc.), tumor size, tumor staging (TNM staging), and pathological type (squamous cell carcinoma, adenocarcinoma, etc.); Preoperative examination indexes, such as routine blood test (white blood cell count, red blood cell count, hemoglobin, and platelet count), coagulation function indexes (prothrombin time, activated partial thromboplastin time, fibrinogen), liver and kidney function indexes (alanine aminotransferase, aspartate aminotransferase, total bilirubin, blood creatinine, and urea nitrogen), and serum proteins (albumin and globulin); Operation-related information, including operation time, bleeding volume during operation, flap type (forearm flap, anterolateral thigh flap, latissimus dorsi flap, etc.), flap resection area, vascular anastomosis method, and whether blood transfusion is required; Postoperative data, such as postoperative hospital stay, whether complications occurred, and the time of complications.

### 2.3 The definition of complications after free flap reconstruction

We know whether the patients have postoperative complications through clinical data. Clinically, the complications after free flap reconstruction include the following Flap necrosis/partial necrosis, Vascular crisis (arterial insufficiency and venous congestion, Infection (flap or surgical site infection), Flap bulkiness or contracture. Patients with one or more of the above symptoms were included in the complication group, and patients without any of the above symptoms were included in the non-complication group.

### 2.4 Statistical analysis

SPSS 26.0 and R 4.5.1 software were performed. Continuous variables conforming to a normal distribution were expressed as mean ± standard deviation (mean ± SD), and intergroup comparisons were conducted using the independent samples *t* test. Categorical variables were presented as frequencies and percentages, and intergroup comparisons were performed using the *χ*² test or Fisher’s exact test. The research subjects were randomly divided into the training set and validation set at a 7:3 ratio using the ‘caret’ package of R software. The statistically significant factors in univariate analysis are included in multivariate analysis, which adopts Logistic regression model and uses random forest algorithm to evaluate the contribution of influencing factors. A nomogram model was constructed using the “rms” package in R software. The predictive performance of the model was evaluated by the receiver operating characteristic (ROC) curve, generated using the “pROC” package, and the concordance index (C-index) was calculated. Internal validation of the model was performed using the Bootstrap method, and calibration curves were plotted to assess model calibration. Decision curve analysis (DCA) was conducted using the “DCA” package to evaluate the clinical utility of the model. A two-sided *P* < 0.05 was considered statistically significant.

## 3 Results

### 3.1 Comparison of incidence of complications and general data between training set and validation set

A total of 341 patients undergoing free skin flap reconstruction for head and neck tumors were included, of which 239 cases in the training set and 102 cases in the validation set. There was no statistically significant difference in the incidence of complications and general data between the training set and validation set (*P*>0.05) in **Table 1**.

**Table 1:** Comparison of general data between training set and validation set.

Index		Training set (n=239)	Validation set (n=102)	*t/χ²*	*P*
Age (years)		59.41 ± 8.53	60.12 ± 8.71	0.699	0.484
BMI (kg/m2)		23.05 ± 3.34	22.98 ± 3.27	0.178	0.856
Gender	Male	136 (56.93)	52 (50.98)	1.014	0.313
	Female	103 (43.07)	50 (49.02)		
Smoking history	Yes	91 (37.96)	34 (33.33)	0.692	0.405
	No	148 (62.04)	68 (66.67)		
Drinking history	Yes	63 (26.36)	21 (20.59)	1.282	0.257
	No	176 (73.64)	81 (79.41)		
Combined hypertension	Yes	46 (19.24)	24 (23.53)	0.803	0.370
	No	193 (80.75)	78 (76.47)		
Combined diabetes	Yes	42 (17.57)	17 (16.67)	0.041	0.839
	No	197 (82.43)	85 (83.33)		
Combined cardiovascular disease	Yes	24 (10.04)	10 (9.80)	0.004	0.946
	No	215 (89.96)	92 (90.20)		
Combined lung disease	Yes	14 (5.86)	5 (4.90)	0.124	0.724
	No	225 (94.14)	97 (95.10)		
Tumor location	Oral cavity	75 (31.38)	26 (25.49)	1.349	0.509
	Throat	84 (35.15)	41 (40.20)		
	Neck	80 (33.47)	35 (34.31)		
Tumor size (cm)		4.35 ± 1.70	4.48 ± 1.65	0.652	0.514
Tumor staging	Stages I-II	106 (44.35)	41 (40.20)	0.503	0.478
	Stage III-IV	133 (55.65)	61 (59.80)		
Pathological type	Squamous cell carcinoma	197 (82.43)	81 (79.41)	0.431	0.511
	Glandular cancer	42 (17.57)	21 (20.59)		
White blood cell count (×10/L)		7.08 ± 2.03	7.15 ± 2.10	0.288	0.773
Red blood cell count (×10^12^/L)		4.59 ± 0.65	4.61 ± 0.64	0.261	0.794
Hemoglobin (g/L)		119.32 ± 14.72	118.95 ± 14.91	0.211	0.832
Platelet count (×10/L)		203.42 ± 33.71	204.15 ± 33.28	0.183	0.854
Prothrombin time (s)		12.25 ± 1.51	12.30 ± 1.49	0.281	0.778
Activated partial thromboplastin time (s)		32.35 ± 4.55	32.50 ± 4.48	0.280	0.779
Fibrinogen (g/L)		3.49 ± 0.86	3.51 ± 0.85	0.197	0.843
Alanine aminotransferase (U/L)		28.12 ± 11.52	27.98 ± 11.65	0.102	0.918
Aspartate aminotransferase (U/L)		26.55 ± 10.23	26.68 ± 10.15	0.107	0.914
Total bilirubin (μmol/L)		15.44 ± 4.75	15.52 ± 4.70	0.142	0.886
Blood creatinine (μmol/L)		85.24 ± 16.33	85.40 ± 16.41	0.082	0.934
Urea nitrogen (mmol/L)		5.62 ± 1.39	5.65 ± 1.37	0.183	0.854
Albumin (g/L)		39.12 ± 4.56	39.08 ± 4.61	0.073	0.941
Globulin (g/L)		29.12 ± 2.63	29.15 ± 2.60	0.096	0.923
Operation time (h)		3.68 ± 1.41	3.72 ± 1.39	0.240	0.809
Intraoperative bleeding volume (ml)		295.32 ± 112.56	301.45 ± 110.87	0.462	0.644
Flap type	Forearm flap	71 (29.71)	26 (25.49)	2.331	0.311
	Anterolateral thigh flap	120 (50.21)	48 (47.06)		
	Latissimus dorsi flap	48 (20.08)	28 (27.45)		
Flap resection area (cm^2^)		98.76 ± 20.45	99.32 ± 20.12	0.232	0.816
Vascular anastomosis method	End-to-end anastomosis	134 (56.07)	50 (49.02)	1.429	0.231
	End-to-side anastomosis	105 (43.93)	52 (50.98)		
Is there a blood transfusion	Yes	106 (44.35)	43 (42.16)	0.140	0.708
	No	133 (55.65)	59 (57.84)		
Postoperative Hospitalization (D)		15.52 ± 3.78	15.65 ± 3.72	0.089	0.928

### 3.2 Comparison of general information between complication group and non-complication group

A total of 239 patients, including 60 cases (25.10%) in the complication group and 179 cases (74.90%) in the no-complication group. The differences in age, hypertension, diabetes, operation time, bleeding volume, flap type, and flap resection area between the two groups were statistically significant (*P* < 0.05) in **Table 2**.

**Table 2:** Comparison of general data between complication group and no-complication group.

Index		Complication group (n=60)	No complication group (n=179)	*t/χ²*	*P*
Age (years)		62.31 ± 8.64	58.56 ± 8.42	2.966	0.003
BMI (kg/m2)		23.45 ± 3.52	22.84 ± 3.15	1.259	0.209
Gender	Male	37 (61.67)	99 (55.31)	0.741	0.389
	Female	23 (38.33)	80 (44.69)		
Smoking history	Yes	26 (43.33)	65 (36.31)	0.939	0.332
	No	34 (56.67)	114 (63.69)		
Drinking history	Yes	17 (28.33)	46 (25.69)	0.160	0.688
	No	43 (71.67)	133 (74.31)		
Combined hypertension	Yes	22 (36.67)	24 (13.41)	15.641	0.013
	No	38 (63.33)	155 (86.59)		
Combined diabetes	Yes	15 (25.00)	27 (15.08)	3.333	0.067
	No	44 (75.00)	153 (85.47)		
Combined cardiovascular disease	Yes	8 (13.33)	16 (8.94)	0.960	0.327
	No	52 (86.67)	163 (91.06)		
Combined lung disease	Yes	4 (6.67)	10 (5.59)	0.001	0.992
	No	56 (93.33)	169 (94.41)		
Tumor location	Oral cavity	17 (28.33)	58 (32.40)	0.481	0.786
	Throat	21 (35.00)	63 (35.20)		
	Neck	22 (36.67)	58 (32.40)		
Tumor size (cm)		4.65 ± 1.74	4.23 ± 1.67	1.668	0.096
Tumor staging	Stages I-II	24 (40.00)	82 (45.81)	0.614	0.433
	Stage III-IV	36 (60.00)	97 (54.19)		
Pathological type	Squamous cell carcinoma	49 (81.67)	148 (82.68)	0.032	0.858
	Glandular cancer	11 (18.33)	31 (17.32)		
White blood cell count (×10/L)		7.23 ± 2.14	7.02 ± 1.98	0.696	0.486
Red blood cell count (×10^12^/L)		4.55 ± 0.67	4.62 ± 0.63	0.733	0.464
Hemoglobin (g/L)		117.45 ± 15.38	120.51 ± 14.48	1.394	0.164
Platelet count (× 10/L)		198.51 ± 35.14	205.25 ± 32.48	1.362	0.174
Prothrombin time (s)		12.34 ± 1.55	12.17 ± 1.47	0.764	0.445
Activated partial thromboplastin time (s)		32.78 ± 4.48	31.97 ± 4.66	1.176	0.240
Fibrinogen (g/L)		3.45 ± 0.88	3.52 ± 0.84	0.552	0.581
Alanine aminotransferase (U/L)		28.46 ± 11.35	27.78 ± 11.74	0.391	0.695
Aspartate aminotransferase (U/L)		26.78 ± 10.46	26.33 ± 9.89	0.300	0.764
Total bilirubin (μmol/L)		15.67 ± 4.79	15.21 ± 4.62	0.661	0.509
Blood creatinine (μmol/L)		85.63 ± 16.77	84.75 ± 15.74	0.368	0.712
Urea nitrogen (mmol/L)		5.66 ± 1.42	5.58 ± 1.35	0.392	0.695
Albumin (g/L)		38.74 ± 5.67	39.38 ± 3.35	1.058	0.291
Globulin (g/L)		28.84 ± 2.71	29.31 ± 2.51	1.230	0.219
Operation time (h)		4.21 ± 1.52	3.69 ± 1.34	2.513	0.012
Intraoperative bleeding volume (ml)		342.46 ± 120.42	299.15 ± 104.95	2.663	0.008
Flap type	Forearm flap	20 (33.33)	51 (28.49)	7.203	0.027
	Anterolateral thigh flap	22 (36.67)	98 (54.75)		
	Latissimus dorsi flap	18 (30.00)	30 (16.76)		
Flap resection area (cm^2^)		106.64 ± 24.62	95.85 ± 18.34	2.599	0.009
Vascular anastomosis method	End-to-end anastomosis	36 (60.00)	98 (54.75)	0.503	0.478
	End-to-side anastomosis	24 (40.00)	81 (45.25)		
Is there a blood transfusion	Yes	32 (53.33)	74 (41.34)	2.618	0.105
	No	28 (46.67)	105 (58.66)		
Postoperative Hospitalization (D)		16.04 ± 4.25	15.34 ± 3.34	1.307	0.192

### 3.3 Logistic regression analysis of complications after free flap reconstruction in patients with head and neck cancer

The occurrence of complications after free flap reconstruction in patients with head and neck tumors was taken as a dependent variable, and the factors statistically significant in univariate analysis were included in the Logistic regression model for multivariate analysis. The results showed that older age, concomitant hypertension, long operation time, large amount of intraoperative bleeding, flap type and large flap resection area were the independent risk factors for complications after free flap reconstruction in patients with head and neck tumors (*P* < 0.05) in **Table 3**.

**Table 3:** Multivariate Logistic regression analysis of complication after free flap reconstruction in patient with head and neck cancer.

Factor	*β*	Standard error	*Wald*	*P*	*OR*	95% CI
Age	0.044	0.020	4.789	0.029	1.045	1.005~1.088
Combined hypertension	3.045	0.625	23.739	0.001	21.002	5.171~71.477
Operation time	0.256	0.128	4.016	0.045	1.292	1.006~1.659
Intraoperative bleeding volume	0.004	0.002	5.258	0.022	1.004	1.001~1.008
Flap type	1.626	0.367	19.611	0.001	5.085	2.476~10.444
Flap resection area	0.018	0.009	4.200	0.040	1.018	1.001~1.036
Constant	-10.196	1.843	30.603	0.001	0.001	

### 3.4 Construction of risk factors for complications after free flap reconstruction in patients with head and neck cancer based on random forest algorithm

**Figure 1:**
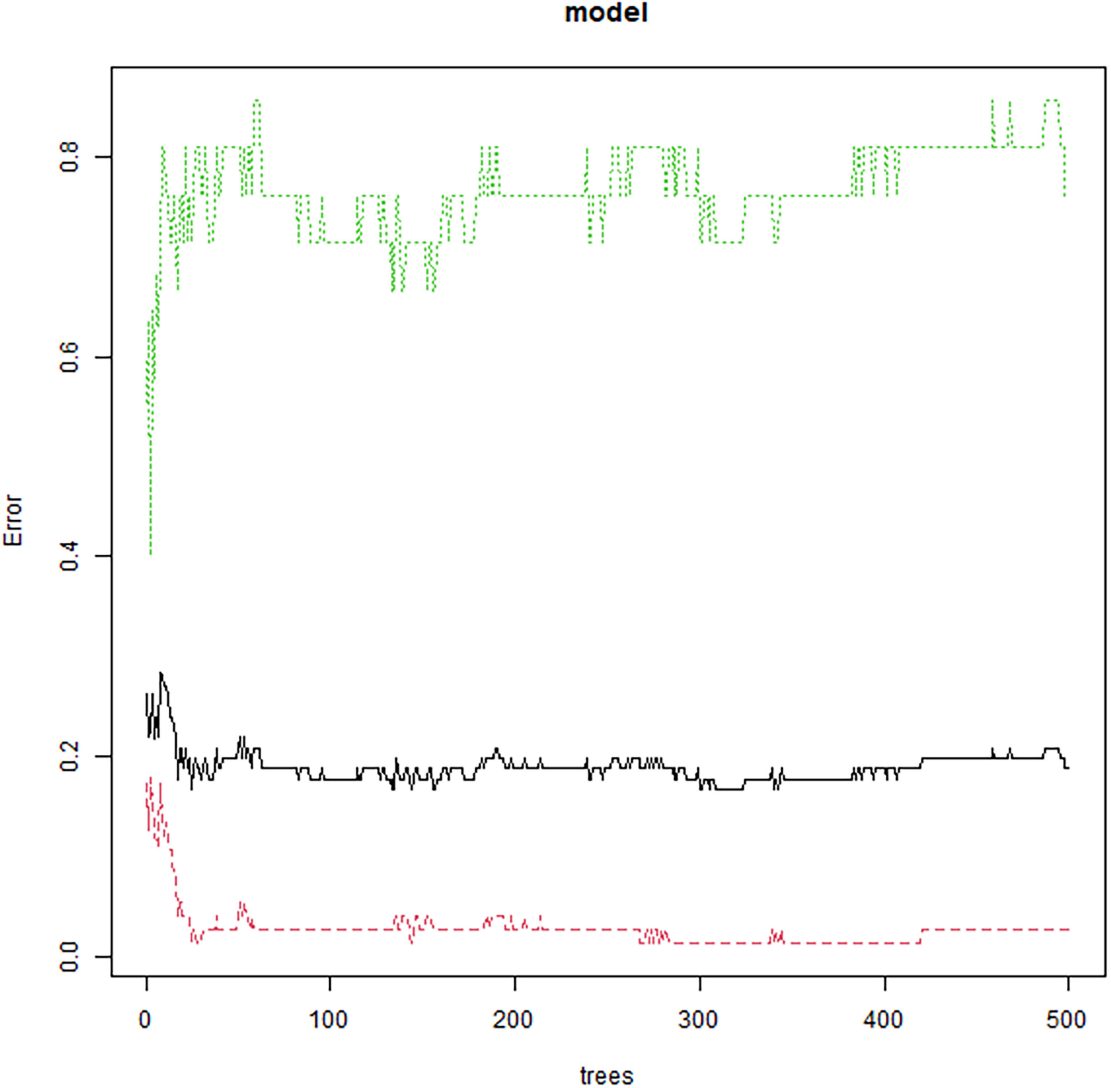
Out-of-bag (OOB) error rate versus number of decision trees in the random forest model (Note: Blue curve represents the overall error rate outside the bag, reflecting the integrated prediction error of the model. Red curve: positive category (complication) bag error rate, reflecting changes in the ability to identify complications. Green curve: Negative class (no complications) bag error rate, showing non complication prediction stability).

**Figure 2:**
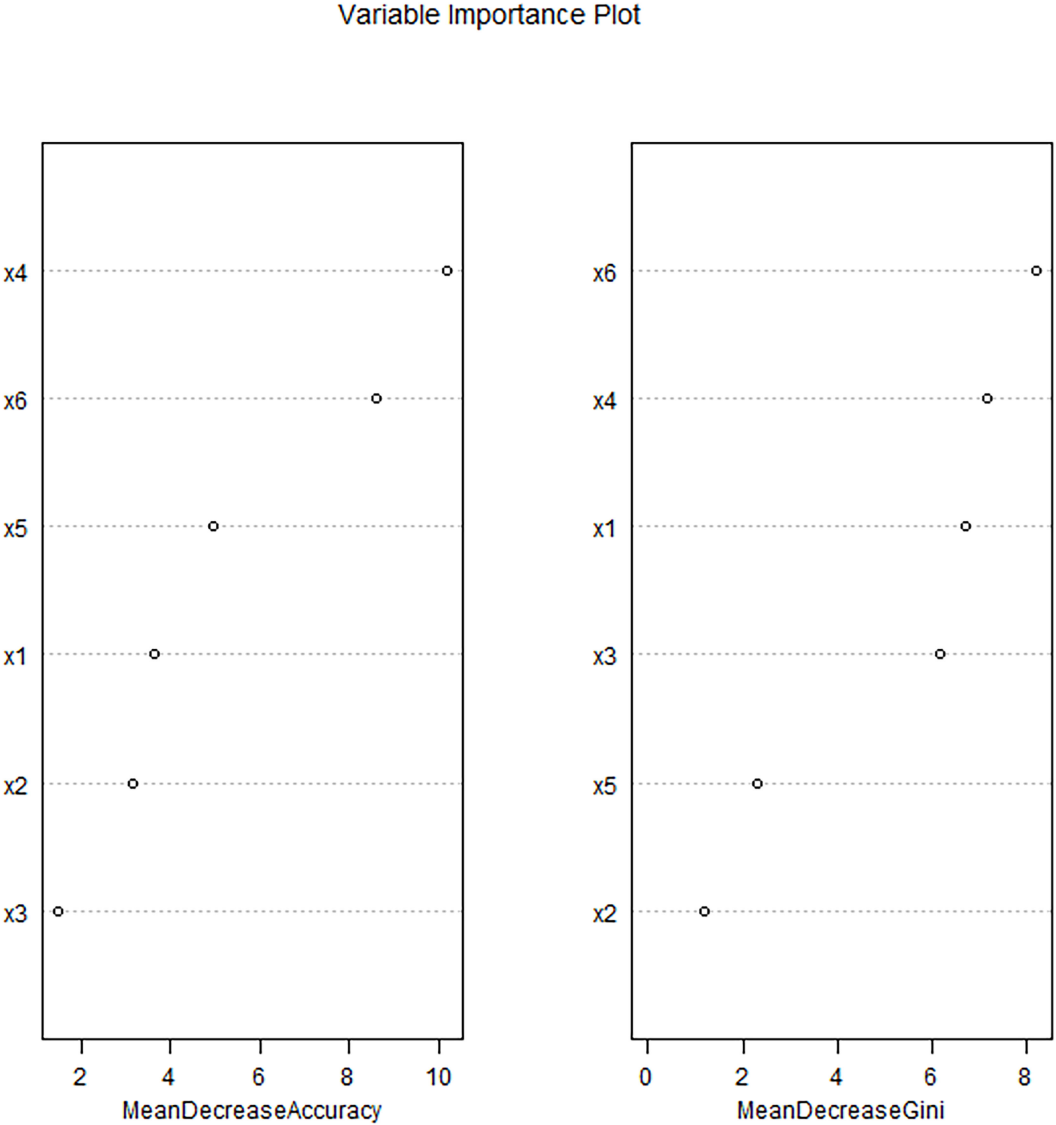
Ranking diagram of importance of influencing factors of complications after free flap reconstruction in patients with head and neck cancer.

The random forest model was constructed with the occurrence of postoperative complications as the dependent variable (No=0, Yes=1), and age, concomitant hypertension, operation time, flap type, intraoperative blood loss, and flap resection area as the independent variables (**Table 4**). The average reduction of the value of Gini of variable was in direct proportion to its contribution in the model. The contribution magnitudes of each variable obtained was flap area, bleeding volume during operation, age, operation time, flap type and complicate hypertension. Multivariate logistic regression showed that flap type was an independent risk factor for complications (*OR* = 12.712, 95% *CI*: 3.320-48.670, *P* = 0.001), indicating a significant independent effect. However, in the random forest model, its importance ranks fifth (after flap area, intraoperative blood loss, age, and surgery time), indicating that although flap type independently increases risk, its predictive contribution in the integrated model may be influenced by the interaction of other intraoperative factors (such as complex flap types often accompanied by large defects). Step-by-step random forest analysis was performed according to the importance ranking of variables. The results showed that the error rate of data outside the bag was the lowest when the number of variables was 6. Therefore, these six variables were included in the random forest algorithm and multi-factor Logistic regression to establish a prediction model (**Figures 1**, **2**).

**Table 4:** Variable assignment table.

Variable	Meaning	Evaluation
X1	Age	Continuous variable
X2	Combined hypertension	No=0, yes=1
X3	Operation time	Continuous variable
X4	Intraoperative bleeding volume	Continuous variable
X5	Flap type	Forearm flap=0, anterolateral thigh flap=1, latissimus dorsi flap=2
X6	Flap resection area	Continuous variable
Y	Are there postoperative complications	No=0, yes=1

### 3.5 Construction and validation of nomogram prediction model

**Figure 3:**
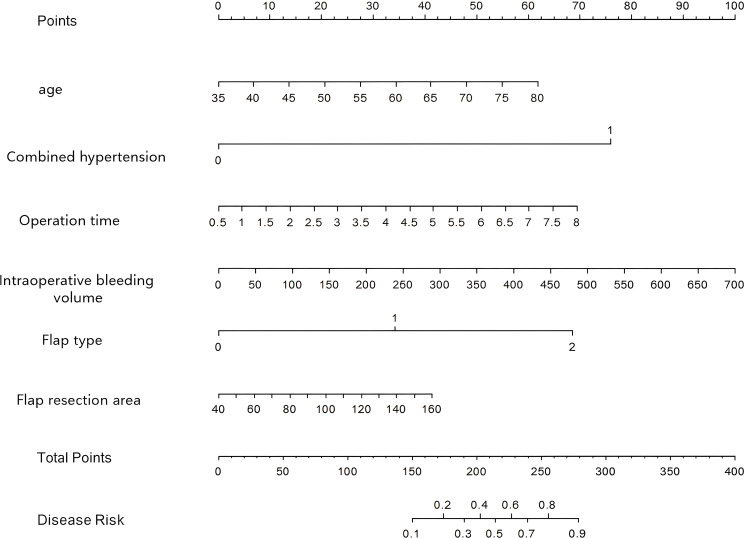
Nomogram prediction model for complications after free flap reconstruction in patients with head and neck cancer.

A Nomogram model was constructed to predict the complications of free flap reconstruction in patients with head and neck cancer. Six independent factors, including age, concomitant hypertension, operation time, flap type, intraoperative bleeding volume and flap resection area were integrated into a nomogram model, which was used to predict the complications of free flap reconstruction in patients with head and neck cancer (**Figure 3**).

**Figure 4:**
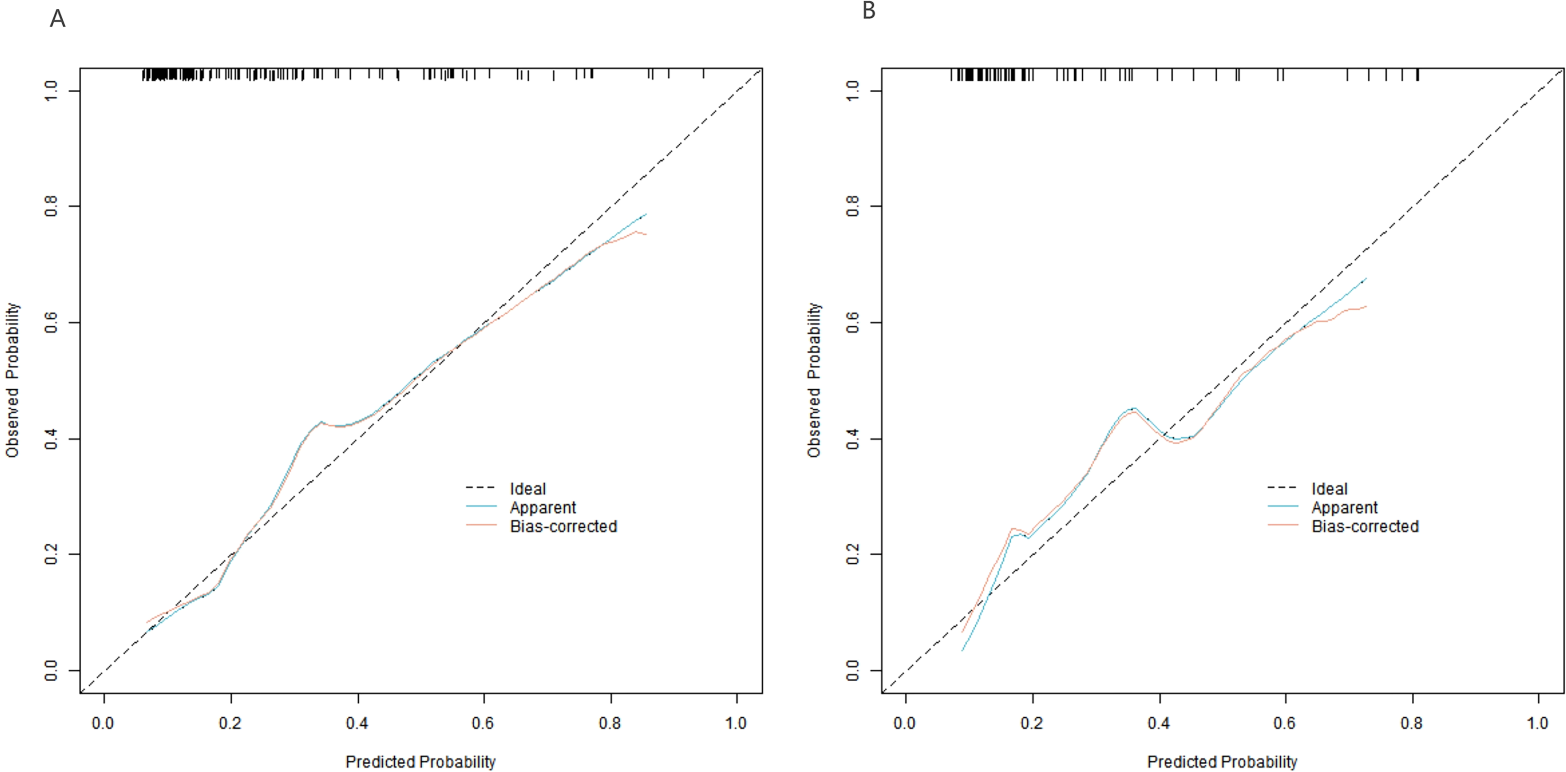
Calibration curve [**(A)** training set, and **(B)** validation set].

**Figure 5:**
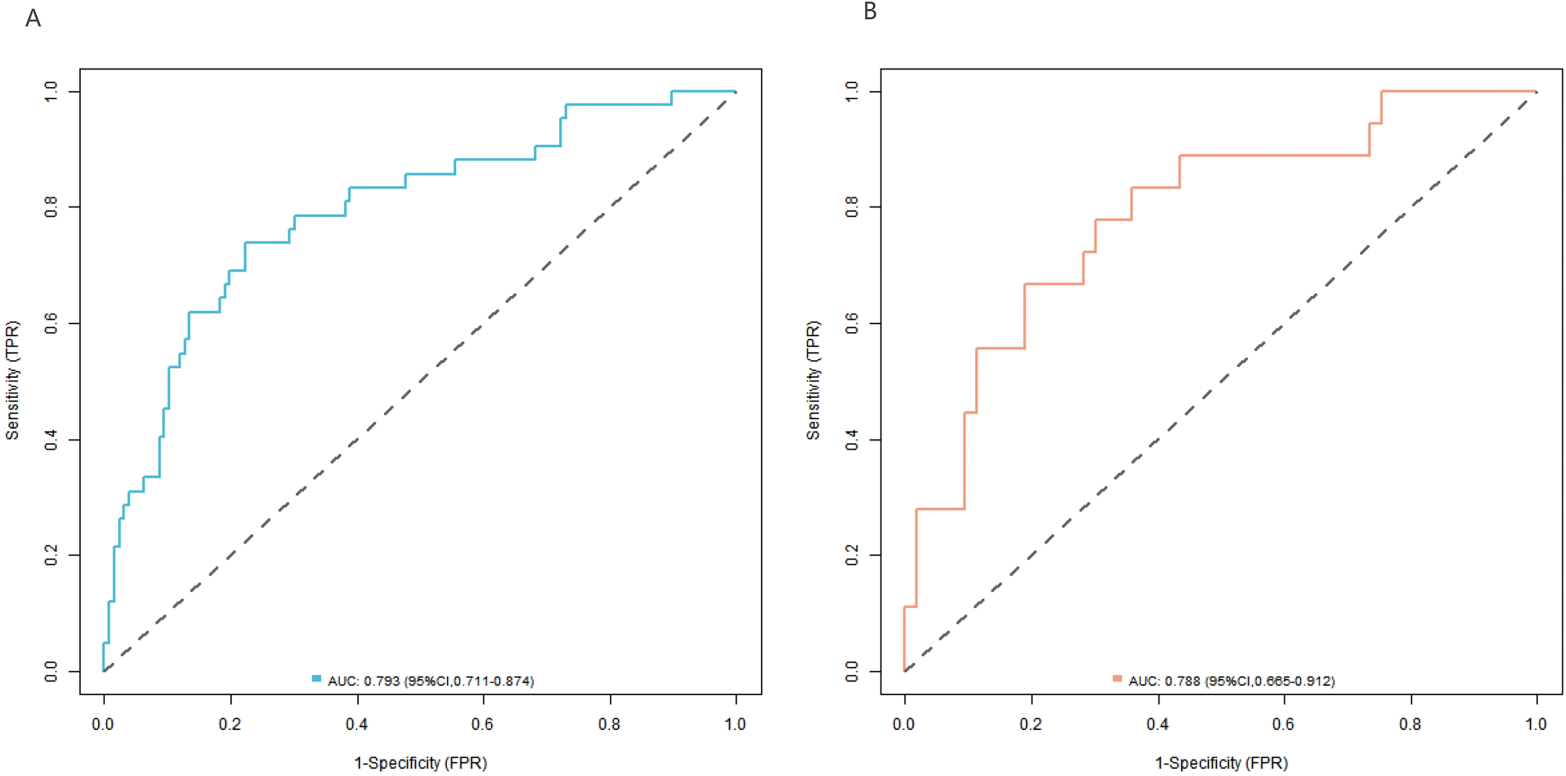
ROC curve [**(A)** training set, and **(B)** validation set].

The C-index of the nomogram model was 0.796 and 0.774 in the complication group and the no-complication group, respectively. The mean absolute error of the calibration curve for the predicted value and the true value was 0.144 and 0.150, respectively. The results of Hosmer-Lemeshow test were *χ*^2^ = 7.004, *P* = 0.536 and *χ*^2^ = 3.102, and *P* = 0.927, respectively. The ROC curves were shown in the training set and validation set. The AUC of the nomogram model for predicting postoperative complications was 0.793 (95% *CI*: 0.711–0.874) and 0.788 (95% *CI*: 0.665–0.912), respectively, and the sensitivity and specificity were 0.778, 0.738, and 0.736, 0.667, respectively, demonstrating that the complication group and the no-complication group had good prediction value. The calibration curve is shown in **Figure 4** and the ROC curve is shown in **Figure 5**.

### 3.6 Analysis of decision curve of nomogram prediction model

**Figure 6:**
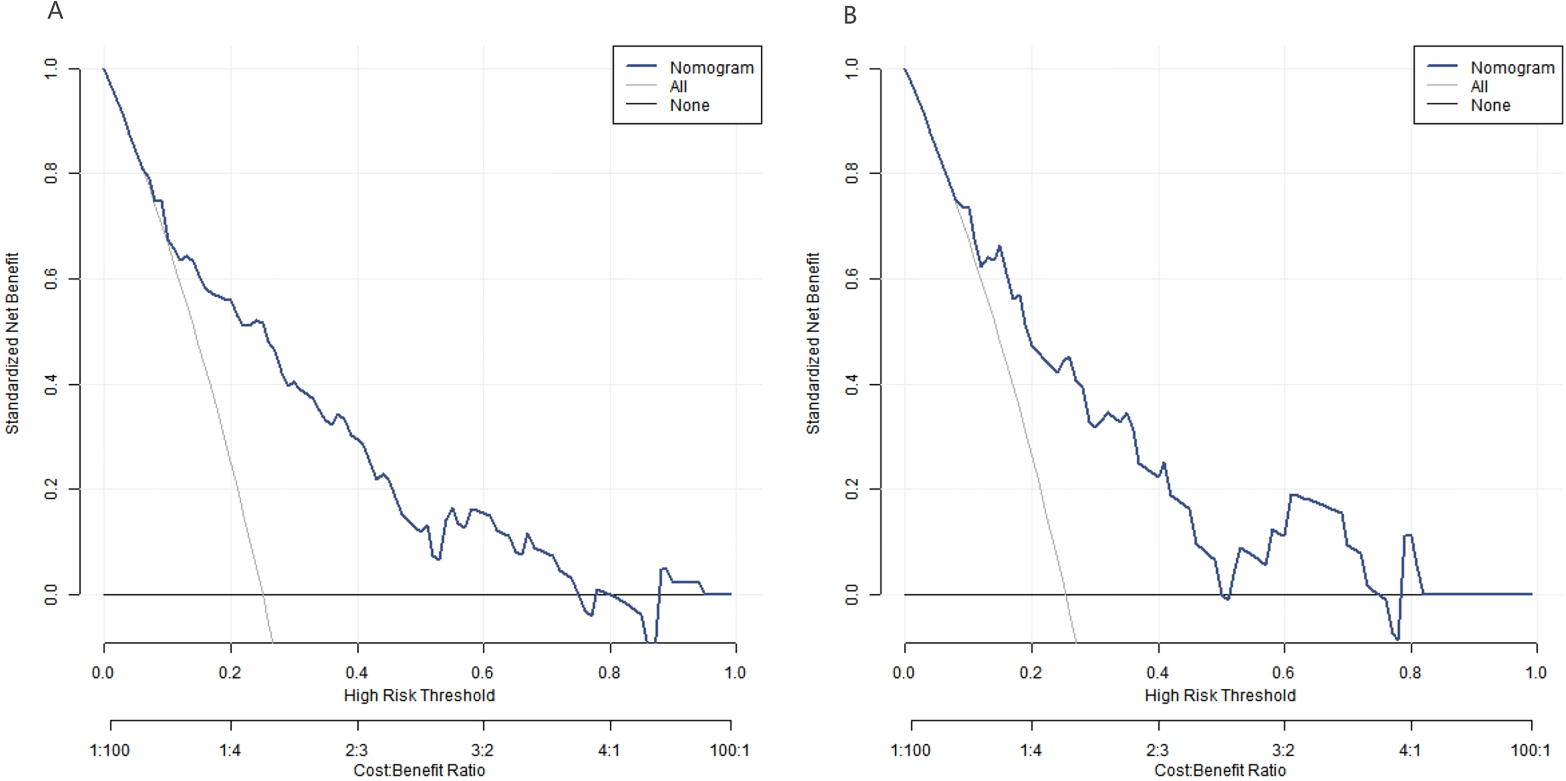
Decision curve [**(A)** training set and **(B)** validation set].

The decision curve showed that when the threshold probability was within the range of 0.10–0.90, the application of the nomogram model constructed in this study to predict the complications after free flap reconstruction in patients with head and neck cancer was more clinically beneficial than the preoperative decision that all complications occurred or none occurred in **Figure 6**.

### 3.7 Model performance across different subgroups of complications

Based on the Nomogram model constructed in the study, validation of the model’s predictive performance was conducted for four core complication subtypes (flap necrosis/partial necrosis, vascular crisis, infection, flap bulkiness/contracture). The **Table 5** presents indicators of the model’s discriminative ability (AUC), calibration (C-index), and clinical utility across each subtype in both the training and validation sets.

**Table 5:** Model prediction performance by complication subtype (Training set n=239, Validation set n=102).

Complication subtype	Dataset	Sample size (n)	AUC (95%CI)	C-index	Sensitivity	Specificity	Calibration curve MAE	Hosmer-lemeshow test (χ²/P)
Flap Necrosis/Partial Necrosis	Training set	28	0.812 (0.725-0.899)	0.805	0.786	0.752	0.132	5.874/0.664
	Validation set	12	0.795 (0.658-0.932)	0.788	0.750	0.731	0.141	4.921/0.766
Vascular Crisis	Training set	16	0.836 (0.751-0.921)	0.829	0.813	0.785	0.128	4.632/0.796
	Validation set	7	0.818 (0.682-0.954)	0.810	0.714	0.768	0.138	3.895/0.869
Infection (Flap/Surgical Site)	Training set	12	0.779 (0.683-0.875)	0.772	0.750	0.726	0.145	6.218/0.623
	Validation set	5	0.763 (0.615-0.911)	0.755	0.600	0.712	0.152	4.357/0.824
Flap Bulkiness/Contracture	Training set	4	0.745 (0.628-0.862)	0.738	0.750	0.703	0.158	7.102/0.525
	Validation set	2	0.728 (0.561-0.895)	0.720	0.500	0.689	0.165	5.283/0.726
Overall Complications	Training set	60	0.793 (0.711-0.874)	0.796	0.778	0.738	0.144	7.004/0.536
	Validation set	22	0.788 (0.665-0.912)	0.774	0.736	0.667	0.150	3.102/0.927

## 4 Discussion

In the field of head and neck tumor treatment, free flap reconstruction is a core technique, but postoperative complications have long posed challenges to improving clinical outcomes. In this study, Logistic regression and random forest algorithm were used to analyze 137 patients. Key influencing factors such as age, concomitant hypertension, operation time, intraoperative blood loss, flap type and flap resection area were successfully identified and a prediction model with good performance was constructed, which has important guiding significance for clinical practice.

The importance of age as an independent risk factor was highlighted in this study. With age, the physiological function of the body gradually declines, and the tissue repair ability and tolerance to surgical trauma are significantly decreased. In elderly patients, the vascular wall is thickened, the elasticity is reduced, and the blood viscosity is increased. These changes make the establishment and maintenance of blood supply of flap after transplantation face greater challenges. In the free flap reconstruction, adequate and stable blood supply is the key to the survival of the flap, while the compromised vascular conditions in elderly patients increase the risk of flap ischemia and hypoxia, which in turn leads to an increase in the incidence of flap necrosis and infection (6). In addition, the immune system of the elderly also weakened, reducing the ability to resist pathogens, the risk of postoperative infection increased. At the cellular level, senescent cells exhibit slower metabolism and decreased proliferative capacity, and cannot efficiently participate in cell regeneration and matrix synthesis in the tissue repair process, further delaying the wound healing process and making postoperative complications more likely to occur (7). Concomitant hypertension plays an important role in the development of postoperative complications of free flap reconstruction for head and neck tumors. Patients with hypertension have their blood pressure elevated for a long time, and their vascular endothelial cells are impacted by high pressure for a long time, which will lead to vascular endothelial damage, changes in the structure and function of the vascular wall, reduction of vascular elasticity, and dysfunction of vasoconstriction and relaxation. Blood pressure fluctuation may be more significant during surgery, especially during the harvesting and transplantation of free flap (8). Such blood pressure fluctuation will further affect blood perfusion at the surgical site, leaving the local tissue of the flap in an unstable blood flow state, and increasing the risk of flap ischemia. At the same time, vascular lesions caused by hypertension may affect the patency of vascular anastomosis and hinder the effective establishment of flap blood supply (9). From the perspective of pathophysiological mechanism, hypertension activates the renin-angiotensin-aldosterone system (RAAS), resulting in aggravation of vasoconstriction and water and sodium retention, further elevating hypertension and affecting microcirculation. After free flap reconstruction, this poor microcirculation state is not conducive to the nutrient exchange of the flap and the discharge of metabolic wastes, thus creating the conditions for the occurrence of complications (10). Operation time is another key factor. A longer surgical time means that the patient’s body is in a state of surgical trauma and anesthesia for a long time. Surgical trauma will trigger a strong stress response in the body, resulting in the release of a large number of inflammatory mediators in the body, such as IL-6 and TNF-α. These inflammatory mediators can interfere with the body’s normal immune function and tissue repair process, increasing the risk of infection. A long period of anesthesia will also have a negative effect on the respiratory, circulatory and urinary systems, for example, causing pulmonary hypoventilation, hemodynamic instability, and urinary retention. These complications may further affect the postoperative recovery of patients and increase the incidence of other postoperative complications (11). In addition, the long operation time is usually related to the complexity and difficulty of the operation, which may mean that the operations of tissue pulling and squeezing are more frequent in the operation process, which will aggravate the tissue damage, affect the local blood circulation and lymphatic return, and is not conducive to the survival of the flap and wound healing (12). The increase of intraoperative bleeding is closely related to postoperative complications. Massive hemorrhage will lead to a sharp decrease in blood volume of patients, causing hypotension and hypoperfusion of tissues and organs. Blood supply of flap will also be seriously affected. Patients may require transfusion therapy to maintain blood volume, but there are risks associated with the transfusion process itself, such as transfusion reactions, infection with blood-borne diseases, and so on, all of which increase the postoperative risks for patients. Intraoperative bleeding can also lead to blood stasis in the local tissue space and the formation of hematoma (13). Hematomas not only compress the surrounding tissue and affect local blood circulation, but also provide a good medium for bacterial growth and increase the risk of infection. From the perspective of coagulation function, massive hemorrhage may activate the coagulation system of the body, trigger disseminated intravascular coagulation (DIC) and other coagulation disorders, further affect the flap blood supply and tissue repair process, and lead to the occurrence of postoperative complications (14). The type of flap is important in the occurrence of postoperative complications. Different types of flap have different anatomical structures, vascular distribution and tissue characteristics, which will affect the survival rate of the flap and the incidence of complications. For example, the forearm flap is relatively thin in texture and has a relatively short vascular pedicle, which may be relatively difficult to establish in blood supply after vascular anastomosis and flap transplantation. In particular, under the conditions of relatively difficult surgical operation or poor vascular conditions of patients, complications such as blood supply disorder are more likely to occur (15). The anterolateral thigh flap has a large area, which is advantageous in the repair of large tissue defects. However, due to the relatively complex harvesting process, it may cause large damage to the donor site, and the risk of complications in the donor site after surgery is relatively high. The latissimus dorsi flap has thick muscle tissue, which is suitable for repairing some parts that need to be filled with a large amount of tissue. However, due to the large amount of tissue and relatively high demand for blood supply, it also faces certain challenges in the aspects of vascular anastomosis and blood supply establishment, and the incidence of postoperative complications such as flap bloating and local effusion may be higher (16). Therefore, in clinical practice, it is of great importance to reasonably select the type of flap according to the specific conditions of patients, such as tumor location, defect size, and vascular conditions. The area of flap resection is also an important factor affecting postoperative complications. A larger flap cut area means a largerSurgical trauma and longer operation time will increase the physical burden and surgical risk for patients. From the perspective of tissue repair, it is more difficult to establish the blood supply and nutrition supply of large-area flap (17). The survival of the flap depends on an adequate blood supply and good nutrient exchange. However, after large-area flap transplantation, the blood supply to the edge and deep tissue may be relatively insufficient, leading to ischemia and necrosis. In addition, after large-area flap resection, the donor site is also more seriously injured, and complications such as poor healing, infection, and cicatrix hyperplasia may occur. These donor site complications also affect the overall recovery of patients, and increase their hospital stay and medical costs (18).

The importance ranking of variables obtained by the random forest model further verified the importance of these factors and provided a reference for clinical practice on the relative importance of different factors. The flap resection area tops the list, highlighting its key role in affecting postoperative complications, and suggesting that clinicians should carefully evaluate the size of the flap needed in surgical planning, and try to reduce the flap resection area to reduce the risk of complications while ensuring the repair effect (19). Intraoperative bleeding volume and age are immediately following, re-emphasizing the importance of controlling intraoperative bleeding and attention to the specific physiological conditions of older patients. Factors such as operation time, flap type and concomitant hypertension also occupied important positions in the ranking, suggesting that clinicians should optimize the operation procedures, improve operation skills and shorten the operation time during the operation. In order to reduce the postoperative complications, the types of flap should be reasonably selected according to the specific conditions of patients and the management of blood pressure in patients with hypertension should be strengthened (20). While Jones et al. (2) reported preoperative malnutrition as critical, its non-significance in our study likely reflects an insufficient sample size (only 15% had albumin records) rather than biological irrelevance. Similarly, the non-significance of tumor stage (*P* = 0.531) contrasts with Tzelnick et al. (3), possibly due to our exclusion of advanced cases unsuitable for free flap reconstruction. The high OR for flap type in logistic regression indicates its inherent biological risk (e.g., perfusion challenges in musculocutaneous flaps), while the lower ranking in random forest suggests that its effect is context-dependent and attenuated when flap area and bleeding volume are considered, which is consistent with Kreutz-Rodrigues’ (14) findings that flap-related risks are mainly reflected in operative metrics.

The Nomogram model constructed in this study showed good performance in the analysis of calibration curve, ROC curve and decision curve. The calibration curve showed that the predicted value was in good agreement with the actual value, indicating that the model could accurately predict the probability of postoperative complications of patients, and providing a reliable reference for clinicians. The AUC value of ROC curve was high, indicating that the model had good discrimination, and could effectively distinguish patients with and without complications, which was of great value in clinical diagnosis and risk assessment. Decision curve analysis showed that within a certain threshold probability range, there were more clinical benefits using the model for decision-making, further proving the clinical practicality of the model, and helping clinicians to formulate personalized treatment plan according to the model prediction results in the treatment decision-making process, such as strengthening postoperative monitoring for high-risk patients, and taking more active preventive measures, thereby improving the treatment effect and reducing the incidence of complications (21, 22).

However, there are several inherent limitations in this study, which are worth discussing. Firstly, this study is a retrospective study. Confounding factors cannot be controlled through random grouping, and there may be selection bias and information bias. Secondly, the lack of external validation is mainly due to the retrospective nature of the study and the limited availability of multi-center clinical data in the reconstruction of free flaps for head and neck cancer patients at that time. In future studies, multi-center data should be collected for external validation to further improve the model and improve its applicability and reliability in different clinical environments. In addition, this study has the problem of a relatively small sample size, which may reduce the statistical power of detecting clinically significant associations. Although we adopt strict cross-validation techniques to reduce overfitting, the universality of the nomogram still needs to be further verified in a larger-scale multicenter cohort. Fourthly, our study analyzed complications as a composite endpoint. While this provides an overall risk assessment, it does not delineate how the identified risk factors might differentially influence specific types of complications, such as flap necrosis, infection, or vascular crisis. A subset analysis based on complication type could yield more precise insights for targeted prevention strategies but was beyond the scope of this initial model-building study. Future research with larger sample sizes could explore these specific relationships.

In this study, Logistic regression and random forest algorithm were used to deeply analyze the influencing factors of complications after free flap reconstruction in patients with head and neck cancer, and the prediction model was successfully constructed and verified. Age, concomitant hypertension, operation time, bleeding volume during operation, flap type and flap resection area were identified as independent risk factors, and the random forest model clarified the importance ranking of these factors. The constructed Nomogram model has good prediction efficiency and clinical application value, and can provide clinicians with a powerful tool for preoperative risk assessment and prevention of postoperative complications. However, due to the lack of external validation, the model needs to be further improved based on multi-center data in future studies in order to better serve the clinical practice, optimize the therapeutic effect of free flap reconstruction for patients with head and neck tumors, reduce the incidence of postoperative complications, and improve the quality of life and survival rate of patients. This research result provides an important reference for clinical decision-making and research development in the field of head and neck tumor surgery, and is expected to promote further breakthroughs in the management of postoperative complications in this field.

## Data Availability

The raw data supporting the conclusions of this article will be made available by the authors, without undue reservation.
